# Gender difference in functional activity of 4-months-old infants during sleep: A functional near-infrared spectroscopy study

**DOI:** 10.3389/fpsyt.2022.1046821

**Published:** 2023-01-18

**Authors:** Kai Wang, Xiang Ji, Ting Li

**Affiliations:** ^1^Institute of Biomedical Engineering, Chinese Academy of Medical Sciences and Peking Union Medical College, Tianjin, China; ^2^College of Computer Science, Chongqing University, Chongqing, China

**Keywords:** gender difference, functional near-infrared spectroscopy (fNIRS), infants, power spectral density, cerebral cortex oxygenation, brain activation

## Abstract

Sex differences emerge early in infancy. A number of earlier studies have investigated the resting-state network of infant sleep states, and there have been many studies using functional near-infrared spectroscopy (fNIRS) to examine the effects of infant language learning on changes in oxyhemoglobin and deoxyhemoglobin levels. However, studies examining sex differences from the resting-state network of infant sleep states are scarce. This study uses an open access dataset of task-free hemodynamic activity in 4-month-old infants during sleep by fNIRS, to identify some difference between male and female infants. We used Power Spectral Density showing at which frequencies the data variation/variance is high. We have also analyzed some gender differences by analyzing the relationship between individual channels, the degree of activation, etc. The results of this study showed that female and male infants had different Power Spectral Density for oxyhemoglobin and deoxyhemoglobin at rest, showing stronger differences at frontoparietal network, somatomotor network, visual network and dorsal network. This may be due to the differences in the timing or extent of development of those networks. These differences will provide some assistance in future studies of the early education of male and female infants.

## 1. Introduction

From birth to early adulthood, the development of cognitive, emotional, and social functions changes with progressive alteration of brain structure. Among these changes, many gender differences are found. Rationally, such differences occur in the language learning, such as early gender differences in the use of language functions by preschoolers (1), and in the reading abilities of students from kindergarten to fifth grade (2). Gender differences also emerge in the expression and self-regulation of emotions in early infancy (3) and in problem behavior (4).

Some of these findings can be derived from an analysis of the functional activity of brain regions that can be studied by resting states or task-free functional connectivity (FC) (5). There are two methods to measure hemodynamic responses to explain task-free functional connectivity. They are functional near-infrared spectroscopy (fNIRS) (6–8) and functional magnetic resonance imaging (fMRI). However, fMRI has several disadvantages that have a significant impact on infant studies. First, fMRI scanners generate a lot of noise, and this noise can also be distracting to the infants being studied. Second, all fMR scans have safety concerns (e.g., hearing protection can damage the auditory system when inadequate). Thirdly, fMRI recordings require strict head stabilization. There have been studies that point out the principle flaws of fMRI (9).

Compared to the other two neuroimaging techniques more commonly used in infant studies (i.e., EEG and functional MRI), fNIRS offers various practical advantages (10). The principle of fNIRS for functional brain imaging is similar to that of fMRI, which mainly utilizes the differential absorption properties of oxyhemoglobin (HbO), deoxyhemoglobin (HbR) and total-hemoglobin (HbT) (11) in brain tissue for different wavelengths of near-infrared light from 600 to 900 nm to detect the hemodynamic activity of the cerebral cortex in real time and directly. Compared to fMRI (12), fNIRS can measure real-world conditions, improving the accuracy of interpreting brain activation (13). Alternative measures of functional brain activation can be revealed by measuring changes in the relative concentrations of HbO and HbR in sampled areas. These changes reflect the vascular responses associated with neural activity. For example, one study looked at children aged 3 years and older, and they found several brain regions associated with autism spectrum disorders. There is early academic literature using fNIRS to examine sex differences, many of which are examining the effects of language learning on changes in HbR and HbO levels, such as infant spoken sign language activation (14), the role of the left inferior frontal cortex in infant audiovisual speech perception (15). The fNIRS acquisition of infant datasets studied in this paper has also been well established in the academic community, including studies on the removal of motion artifacts from the acquired data (16), and studies on the pre-processing of these data (17), which provide a good direction for future data processing. There are also studies using fNIRS to analyze infant and child brain activity, such as the study on the local brain function activity of infants watching adult social activities (18) and using fNIRS to explore the effects of methylphenidate on brain activity during a VR-based working memory task in children (19).

However, due to the difficulty of collecting signals from infants and children, it is difficult to observe a fixed pattern of changes in oxyhemoglobin and deoxyhemoglobin, and the quality of the signals collected is somewhat poorer than that of adults. As a result, there have been fewer studies in this area. Most of these studies, like those studying children, have focused on the effects of the early language environment on changes in HbR and HbO levels and have not thoroughly examined gender differences in early development. In addition, most early infant studies define cortical activation based on HbO signaling only (20) and do not consider HbR signaling either before or after data analysis. However, the fNIRS community has raised awareness of the importance of assessing HbO and HbR (and possibly HbT) in order to draw solid scientific conclusions, and most publications now report results for both parameters.

In addition, the brain mechanism of infant sleep states has also been studied. Infants exhibit spontaneous discrete and cyclic active and quiet sleep patterns (21). Another brain mechanism of another stage in infant sleep, indeterminate sleep, has also been added in a study. This study also suggested that the transition from the active sleep stage to the rapid eye movement stage, which accounts for most of the sleep time, contributes to the regulation of emotions in the developing brain (22). And the presence of resting-state networks in the unsedated infant brain born at full term has been found. They are encompassing sensory cortices, parietal and temporal areas, and the prefrontal cortex (23). There is still much to investigate in these sleep stages, such as whether there are gender differences in resting-state network connectivity and activity in various brain regions during sleep.

We, therefore, hope to fill this gap in the field, and in this paper, we use the fNIRS open dataset of task-free hemodynamic activity during sleep in 4-month-old infants to investigate sex differences in infants. The equipment used for data acquisition in this dataset was an fNIRS headpiece consisting of 16 transmitters and 24 detectors (NIRx NIRScout Medical Technologies, CA, USA). We used Power Spectral Density (PSD), a signal analysis method that converts the time domain signal to the frequency domain, to visualize the variation/variance (energy) as a function of frequency, showing at which frequencies the data variation/variance is high, which may be useful for further analysis. We have also analyzed some gender differences by analyzing the relationship between individual channels, the degree of activation, etc., which we hope will shed light on future research in this area.

## 2. Materials and methods

### 2.1. Dataset

The dataset used in this study is high-quality fNIRS data collected from a large sample of participants, which accurately describes large-scale patterns of functional connectivity at the population level in 4-month-old infants (20). The sample in the dataset consisted of 104 participants who were recorded during natural sleep for relative changes in HbO and HbR in the cerebral cortex. There were 51 female and 48 male infants, the oldest being 131 days old and the youngest 115 days old, and each participant had at least 9 min of prolonged recording. After the infant has been fitted with the fNIRS, the parents help them to fall asleep and the recording begins when the infant shows visible signs of sleep. During the recording period, the parents were asked to be as quiet as possible with minimal movement to improve the accuracy of the recorded data (24).

### 2.2. Data processing

Data preprocessing was performed using third-party programs (e.g., Homer3) and internal scripts from Matlab (R2022a, Math Works, MA, USA) (25). First, we filtered the data collected in the dataset. The actual number of channels analyzed was 46 in total. The fNIRS is composed as follows: 16 Light source and 24 detectors are placed on a flexible cloth EEG-cap (Easycap GmbH, Germany) covering the frontal, temporal, parietal and occipital regions of both hemispheres. Each pair of adjacent light emitters and detectors formed one measurement channel, generating 52 channels for each hemoglobin oxygenation state (i.e., HbO and HbR). The occipital channel was discarded for all participants because the posterior part of the infant’s head rests against the parent’s body during data acquisition, and any slight movement would result in misalignment of these specific optical elements (26) and so be particularly prone to contain signal artifacts. Therefore, data from only the remaining 14 light sources and 19 detectors (i.e., 46 channels) were analyzed. The raw light intensity data were then converted to optical density variations by calculating the negative logarithm of the ratio of the detected light intensity to the reference baseline value (i.e., the average signal). Motion artifacts were removed using a wavelet-based denoising method. After this step, the optical density data were converted into HbO and HbR concentration variations using a modified Beer-Lambert law (27):


$$OD=\frac{I_{out}\left(t,\lambda \right)}{I_{in}\left(t,\lambda \right)}=e^{-D\left(\lambda \right)x\mu_{a}\left(t,\lambda \right)+G\left(\lambda \right)}$$

In the formula, *I*_*in*_ (*t*, λ) denotes the intensity of light entering the medium at moment *t, I*_*out*_ (*t*, λ) denotes the intensity of light detected after passing through the medium at moment t, λ is the wavelength of light, x denotes the distance traveled by light, and μ_*a*_ (*t*, λ) is the absorption coefficient of the medium (e.g., biological tissue). The parameter G(λ) describes the loss of light intensity due to scattering and the differential path length factor D(λ) indicates the increase in distance light travels due to random walking (i.e., X = Dx). the differential path length factor value depends on several factors such as the age of the participant, the wavelength and the type of tissue being evaluated. According to the documentation for the dataset, we set the differential path factors to 5.3 and 4.2 (24). μ_*a*_ (*t*, λ) is given by the specific extinction coefficient e(a) of all the different absorbing substances present in the medium (e.g., water, fat, HbO, and HbR) and their concentrations *C*, such that:


$$\begin{array}{l} \mu_{a}\left(t,\lambda \right)=\varepsilon_{water}\left(\lambda \right)C_{water}\left(t\right)+\varepsilon_{fat}\left(\lambda \right)C_{fat}\left(t\right)\\ +\varepsilon_{HbO}\left(\lambda \right)C_{HbO}\left(t\right)+\varepsilon_{HbR}\left(\lambda \right)C_{HbR}\left(t\right) \end{array}$$

In the experimental setting, media such as water and fat are considered not to change with time, so only changes in HbO and HbR are considered. Then, in order to obtain the amount of change in the concentration of HbO and HbR, we combine the above two equations to obtain the following Equation:


$$\begin{array}{l} \Delta OD\left(\lambda \right)=ln\frac{I_{out}\left(t_{2},\lambda \right)}{I_{in}\left(t_{0},\lambda \right)}-ln\frac{I_{out}\left(t_{1},\lambda \right)}{I_{in}\left(t_{0},\lambda \right)}=-ln\frac{I_{out}\left(t_{1},\lambda \right)}{I_{in}\left(t_{2},\lambda \right)}\\ =Dx(\varepsilon_{HbO}\left(\lambda \right)\Delta C_{HbO}+\varepsilon_{HbR}\left(\lambda \right)\Delta C_{HbR}) \end{array}$$

After we complete the conversion of the original light signal to the change in concentration of HbO, HbR, an interference regression model including temporal filtering and global signal regression was used to attenuate fNIRS signal contamination caused by fluctuations in the global source system. Contribution of those high-frequency physiological noise sources was accounted for by including sine and cosines functions for frequencies above 0.09 Hz in the model for nuisance regression. Very slow frequency fluctuations and signal drift were modeled by including the first fourth order Lejeune polynomial in the design matrix. By adding the Legendre polynomials to the regression model and then also including the average fNIRS signal in the regression model, the hemodynamic processes that commonly occur in tissues inside and outside the brain, which are thought to largely reflect changes in systemic hemodynamics, are removed. This process of converting raw light intensity data into optical density changes and then into HbO and HbR concentration changes is performed using the initial processing code provided in the dataset. All preprocessed data used in this study were obtained from the above mentioned dataset (24).

### 2.3. Data analyze

We first sorted the pre-processed data for all individuals by sex and then averaged the data for male and female infants separately for HbO, HbR, and HbT. For the PSD curve analysis, we first extracted the pre-processed data, sieved the occipital channels, and then averaged the HbO and HbR concentration data for male and female infants separately. These averages were then Fourier transformed and the PSD data were prepared. For the data between individual channels, we used the PSD data of male and female individuals and then separated the different channels and finally performed a paired *t*-test on them.

The preprocessed data were independently subjected to the FC analysis procedure, which was employed for both HbO and HbR. First, as a measure of functional connectivity across channels, paired Pearson correlation coefficients (corr function in MATLAB) were calculated between each channel pair for each participant for the time course of HbO and HbR signals. A FC matrix can be thought of as the Pearson correlation coefficients for channels m and n, which can be used to display the correlation coefficients for all channels. We can see the distinctions between male and female newborns by looking at this matrix.

To determine the oxygenated and deoxygenated phases (hPod) of hemoglobin, the mean phase shift between the HbO and HbR signals for every individual data was determined for every channel (28). For each channel, the HbO and HbR signals were subjected to Hilbert transforms to get the associated instantaneous phase signals. The phase difference signal between HbO and HbR is then obtained by subtracting these signals from one another. The value of the phase difference under each channel (i.e., hPod) is then obtained by calculating the time average of the phase difference for each pair of HbO and HbR channels. For each subject and the entire group, standard and polarity histograms were generated, with each subject contributing 46 data (i.e. number of channels). *T*-tests were used to look into statistical differences in mean hPod values between males and females.

## 3. Results

### 3.1. Activation maps

**FIGURE 1:**
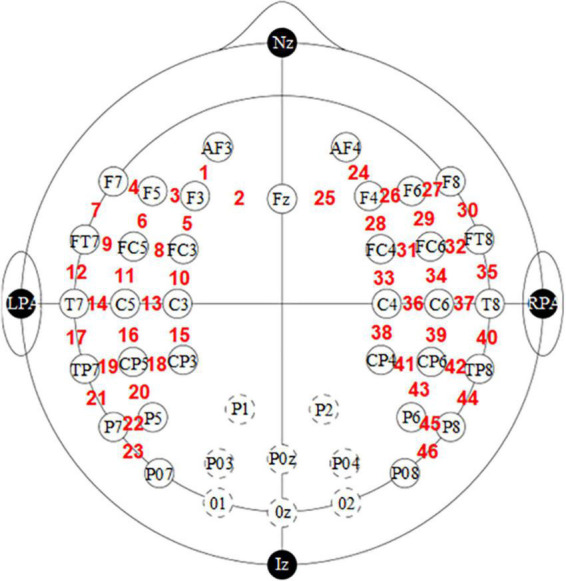
Functional near-infrared spectroscopy optode (sources and detectors with circle in black) and channel (digital in red) localization in the experimental setup. The dotted line indicates the graph with the deleted occipital channel.

We disaggregated the pre-processed data for all people by gender and then averaged the data for male and female infants separately; these data included HbO, HbR and HbT. These mean values were also mapped according to channel locations and then superimposed on a two-dimensional model using a 10–20 system as the background. Figure 1 is plotted using the dataset information about the fNIRS optode layout configuration. The information was acquired using the international 10–20 system. The international 10–20 system is a long-established method originally developed for electrode placement in EEG studies (29). This system uses four reference points (nasion, inion, right and left preauricular points) as a reference to determine the 10–20 standard positions of the scalp sensors. Since this data contains information in only two dimensions, a 2D image is plotted here, intending to illustrate the approximate coverage of fNIRS measurements over the cortex.

**FIGURE 2:**
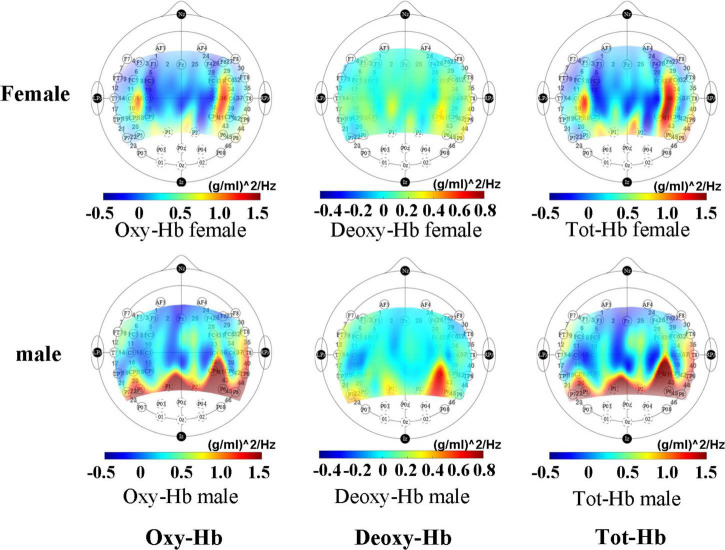
Mean activation maps of female and male infants under non-tasks measured by fNIRS.

From the activation map (Figure 2), it can be seen that male infants have a higher degree of activation in the hindbrain than female infants during sleep. In female infants, brain activation was higher near channel 14 than male infants.

### 3.2. Mean PSD

All research participants’ average HbO and HbR signals were calculated. For log frequency ranges, the mean PSD of the signals was computed and shown.

**FIGURE 3:**
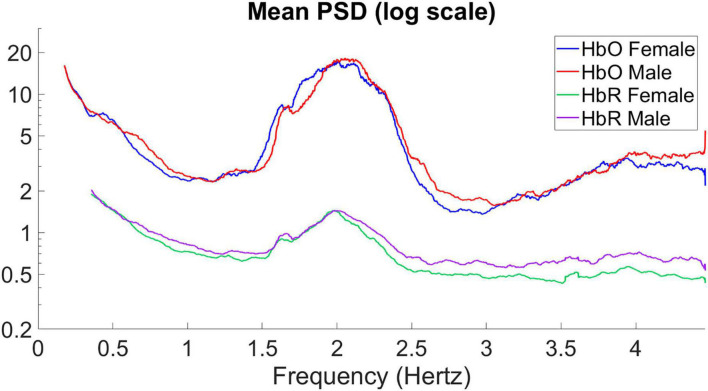
Mean power spectral density of the average signal for HbO and HbR.

To represent the differences between men and women more significantly, we processed the PSDs of the HbO and HbR signals by selecting the higher frequency part of the variance, logarithmically processing the power energy characterized by the *y*-axis, and smoothing the curves to obtain Figure 3.

**FIGURE 4:**
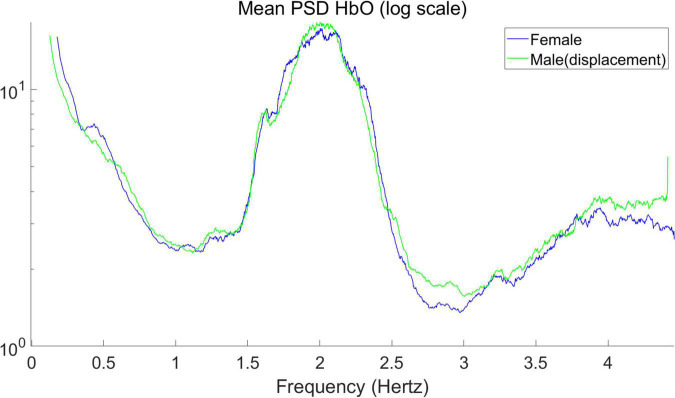
Mean power spectral density of the average signal for HbO (after displacement in Figure 3).

The mean PSD of the signals for male and female infants is calculated separately in Figure 3, giving the average power spectral density in the frequency range of 0–4.5 Hz. In hyperbaric oxygen, clear peaks can be observed around 2 and 4 Hz, representing the heart pulse and its harmonics. Higher values are observed at 0–0.5 Hz, which may indicate a respiratory component. The sampling frequency was 15.625 Hz. For the mean PSD, the difference between males and females was more pronounced in the higher frequency band (2.5–4.5 Hz), with higher PSD values in male infants than in female infants for both HbO and HbR, with the difference being particularly pronounced for HbR. In addition, it can be observed that the average power spectral density of HbO of male infants seems to be “lagged” compared with that of female infants. Therefore, we shifted the curve of the male by 0.05 Hz to the left to obtain the result in Figure 4. As you can see, after moving the image, the part below frequency 2.5 overlaps.

### 3.3. Significance tests for individual channels

**FIGURE 5:**
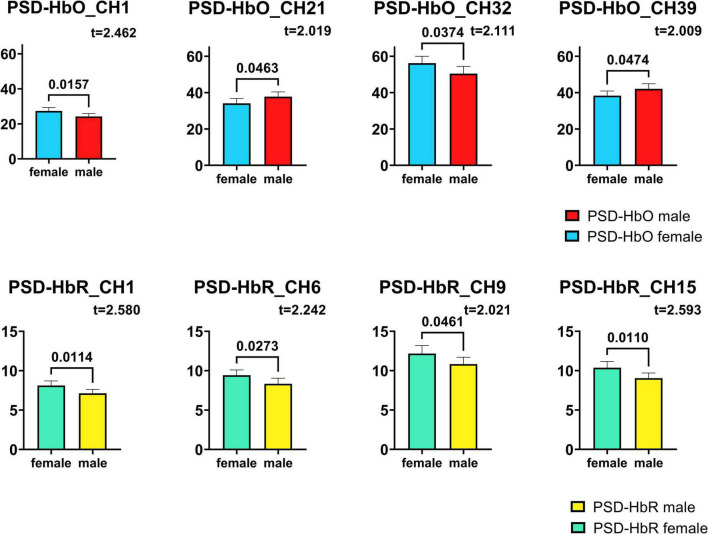
Channels with significant differences in HbO, HbR PSD obtained by *t*-test. The numbers on the link line are the *p*-values in the *t*-test, and the *t*-values are also labeled in the figure.

The PSD of HbO of each Channel was tested by *t*-test, and 4 channels that passed the *t*-test were screened, among which channel 1, channel 21, channel 32, and channel 39 showed the greatest difference between male infants and female infants. The results of the statistics are shown in Figure 5. The PSD signal processing for HbR was also the same for each channel, and 4 channels passed the *t*-test for significant difference, with the largest differences between male and female infants for channel 1, channel 6, channel 9 and channel 15.

The numbers on the link line in the figure are the *p*-values in the *t*-test, and the *t*-values obtained during the *t*-test are also labeled in the figure. All eight channels obtained are significantly different between males and females (*p* < 0.05), which means that with 95% probability, it can be said that the PSD levels of male and female infants are significantly different in these channels.

### 3.4. Correlation adjacency matrix analysis for each channel

**FIGURE 6:**
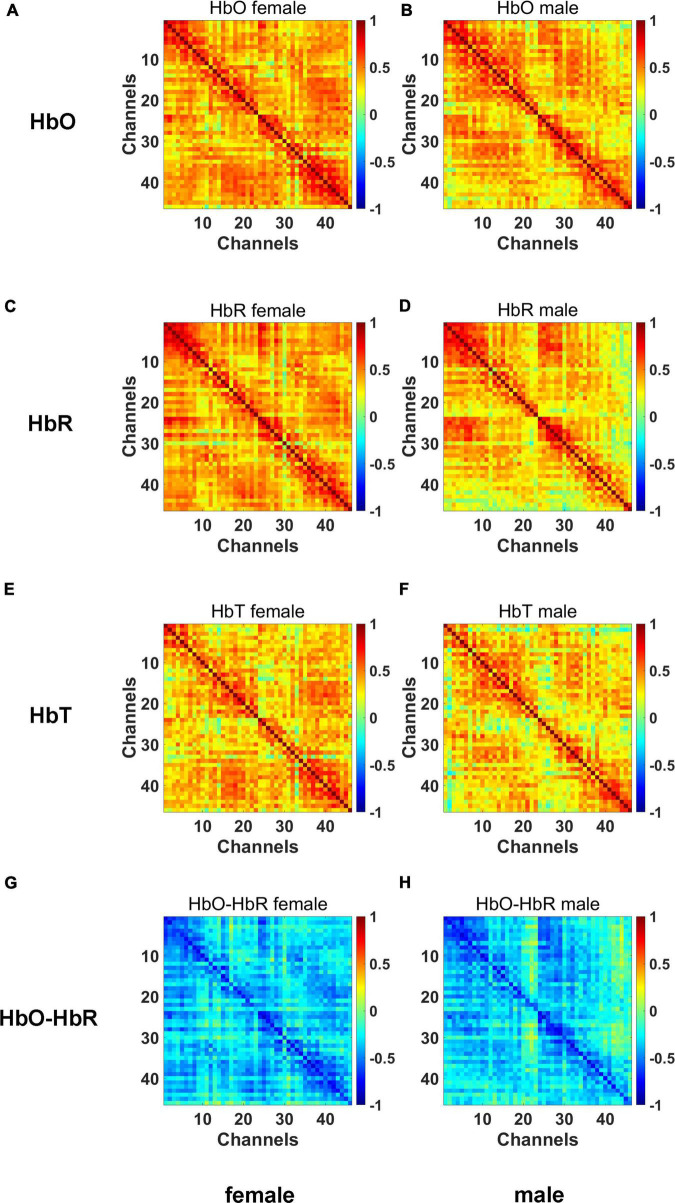
The adjacency matrices for HbO **(A,B)**, HbR **(C,D)**, HbT **(E,F)**, HbO-HbR **(G,H)** concentration data.

The correlation between the HbO, HbR, and HbT concentration values was calculated for each channel and this adjacency matrix was plotted in Figure 6. These adjacency matrices were used to observe the connectivity between the different channels.

The major diagonal of these matrices has the strongest correlation values between neighboring channels, while homotopic channels also exhibit somewhat higher correlation values. We can see the connection between the left and right hemisphere isotopes by the diagonal line from (1,27), (2,28) up to (26,52) in the matrix. The major diagonal of adjacency matrices is symmetric, as should be noted.

Graph (G) and (H) in Figure 6 represents the HbO-HbR adjacency matrices. The pairwise Pearson’s correlation coefficients between the HbO and HbR signals may be used to calculate the adjacency matrix, which can be used to assess the predicted negative correlation between the HbO and HbR time series. Similar to this, there should be a clear phase difference between the HbO and HbR chromophores that is almost antiphase (180°) (28).

These matrices show the anticipated negative correlation as well as a striking resemblance to the patterns seen in the HbO and HbR adjacency matrices. And for the phase difference between HbO and HbR, values close to 180° are indeed revealed.

As can be seen from the connectivity matrix of the channels above, there are some differences between males and females in terms of channel connectivity. The male infants have a somewhat poorer overall connectivity than the female infants, more pronounced at channels 40–46.

**FIGURE 7:**
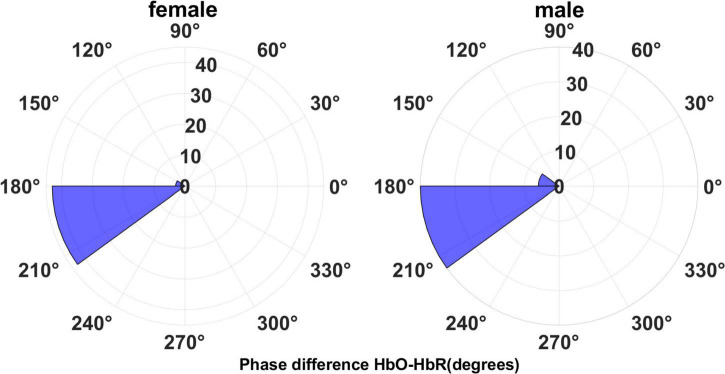
The phase difference for HbO-HbR concentration data.

Figure 7 shows the angle histogram plots of the phase difference between HbO and HbR time series.

**FIGURE 8:**
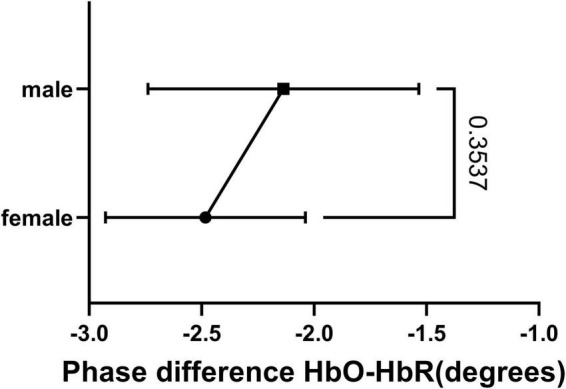
Result of *t*-test of HbO-HbR phase difference between female and male.

The HbO-HbR phase difference in both sexes was mostly distributed in the range of 180°–210°, with a small number distributed in the range of 150°–180°. We performed a *t*-test on the phase-difference HbO-HbR data for males and females and the results are shown in Figure 8. It can be seen that there is no significant difference in the phase difference HbO-HbR between males and females. This makes sense, as the difference between HbO and HbR should have been around 180° from the *a priori* event (28, 30), and this property should be the same for both males and females.

## 4. Discussion

This study shows differences in the levels of HbO and HbR by gender, which were confirmed by analyzing data from publicly available datasets collected using fNIRS. In the resting state without a task, men and women showed different levels of active brain responses. This result does not replicate previous reports, or rather, there is less research in this area in the academy and the focus of this study is more innovative. Previous studies have identified many gender-specific manifestations of cognitive activity (31–35), and there have also been analyses of gender differences in prefrontal cortex hemodynamic responses (36, 37), which goes some way toward demonstrating the reliability of our study.

The participation of infants and young children in experiments might be difficult. They have irregular behavioral patterns, poor attention spans, and an inability to follow directions. When brain imaging is employed, additional challenges appear. Infants are unable to be taught to sit still, do not tolerate headgear well, and have trouble having their hair combed or adjusted in place. High-frequency noise, significant and frequent motion artifacts and other issues with the measured signal might result from these issues. Therefore, while processing and analyzing data, these data quality concerns must be taken into account. Having a some what high-quality data set is not a simple endeavor since data quality in research on newborns is often substantially lower than in studies of adults.

Outside of these areas of research, fNIRS has become an important neuroimaging tool, helping to answer many long-standing questions about infant cognition and providing converging evidence for previous behavioral findings (38). Early fNIRS studies in healthy infants sought to demonstrate the ability of this technique to detect functional brain activation for simple visual (e.g., reverse checkerboard) (39, 40) and auditory stimuli (e.g., music, tones). In the area of object processing, a series of studies have shown that the features used to identify objects are age-dependent, with infants under 9 months of age using only shape information, while older infants rely on color and shape information for object differentiation. These studies also confirm the primary role of the anterior temporal cortex in object recognition, as this region responds selectively during events in which infants are able to notice changes in the characteristic properties of object stimuli (e.g., shape, color), but does not activate under control conditions in which objects remain unchanged. In addition, another fNIRS study showed that the ability to categorize color perception in the visual cortex may already be present in infants aged 5–7 months. Similarly, one study used fNIRS to study number detection in 6-month-old infants, when the infant’s ability to represent numbers mentally is not fully established (41). At this age, the right inferior parietal sulcus showed functional specialization for number perception, which is consistent with the involvement of bilateral parietal regions observed in studies assessing number processing in adults and older children.

In the last decade or so, more and more cognitive neuroscientists have shifted their focus from isolated “regions” to larger “networks.” A study proposes an end-to-end deep learning framework called EEG channel active inference neural network to handle the classification of electroencephalogram-based motor imagery (42). Regarding the results obtained in subsection 3.3 “Significance tests for individual channels,” in conjunction with this one study, we suggest that the differences in HbO may be due to differences in the timing or degree of development of the frontoparietal network (CH1), somatomotor network (CH32, 39) and the visual network (CH21) in male and female infants (42, 43). For the HbR differences, we suggest that the differences may be due to differences in the timing or extent of development of the frontoparietal network (CH1), somatomotor network (CH9, 15), and the dorsal network (CH6) (42, 43).

In summary, this study combines the experience of previous authors and our own understanding to explore the differences in brain hemodynamics during resting state in some male and female infants. These results are unexplored by the current academic community and we hope that our study will shed some light on them for others to follow.

Although fNIRS is relatively resilient in dealing with motion artifacts compared to other neuroimaging techniques, this one advantage can also only be asserted if the optical probe is firmly attached to the head. However, this is more difficult to achieve when testing on infants. Therefore, a thorough data quality assessment of individual data sets is recommended in order to prevent negative effects on the results and interpretation of the study. It is also necessary to work out reliable data processing procedures or to use existing reliable procedures to deal with motion artifacts and physiological noise. It should be noted that this study has limitations. On the one hand, most of the present study only lists the differences found, but does not give much explanation for the causes of these differences, which would require more research time and effort. On the other hand, the entry point of this study was mainly in terms of PSD and less in other areas, so more attention could be paid to additional aspects, such as mean time series amplitude, in future studies.

## 5. Conclusion

To our knowledge, this is the first study to use hemodynamic to explore sex differences in resting-state functional connectivity of the brain during early human development. The results of this study show that male infants have different PSDs for HbO and HbR at rest, showing stronger differences at channels 1, 21, 32, 39, 6, 9, and 15. This may be the result of differences in the timing or extent of development of the frontoparietal network, somatomotor network, visual network, and dorsal network. In terms of channel connectivity, male infants were less well connected overall than female infants. In contrast, there were no significant differences between male and female infants in terms of phase differences between HbO-HbR. These findings suggest that gender differences do manifest themselves in the resting-state functional connectivity of the brain early in human development. These differences will provide some assistance in future studies of the early education of male and female infants.

## Data availability statement

The datasets presented in this study can be found in online repositories. The link of the repository is as follows: https://github.com/ATQlove/GD-infants-fNIRS.

## Ethics statement

The studies involving human participants were reviewed and approved by the Local Ethical Committee at the Basque Center on Cognition, Brain and Language (Donostia—San Sebastián, Spain). Written informed consent to participate in this study was provided by the participants or their legal guardian/next of kin.

## Author contributions

KW was responsible for data analysis and manuscript writing. XJ was responsible for manuscript language and polishing. TL was responsible for manuscript review. All authors contributed to the article and approved the submitted version.
